# Supplementary material for: Patterns of Intron Gain and Loss in Fungi
Source: PLoS Biol. 2004 Nov 30;2(12):e422. doi: 10.1371/journal.pbio.0020422 (PMC532390; doi:10.1371/journal.pbio.0020422)
Supplement: Table S1 — Also available at http://genes.mit.edu/NielsenEtAl/. (4.3 MB ZIP). [file pbio.0020422.st001.zip › NielsenEtAl/html/546.html]

AN2212.1.NCU01225.1.MG05478.1.FG02029.1


```
 CLUSTAL W (1.82) Multiple Sequence Alignments - Introns Inserted


Sequence 1: NCU01225.1	235 aa
Sequence 2: MG05478.1	239 aa
Sequence 3: FG02029.1	234 aa
Sequence 4: AN2212.1	246 aa
Alignment Length: 247 aa
Number Identitical Residues: 116 aa
Alignment Score (without introns) 5636


MG05478.1 	MAMSSRLRRVAKELQDIQNDRDNSGVMASAINESDLTHLRGSFPGPPGTPYEGGTYVVDI
NCU01225.1	MATT-RERRVAKELQDIWNDRETSGVMAEPLDSSNLSHLKGSFSGPPDSPYAGGTYEVDI
FG02029.1 	--MS-RDRRIMKELADIQHDHDSSGVNAILVSEGNMTHLKGSFPAPPDTPYSGGTYTIDI
AN2212.1  	MASN-RIRRIAKEIADIRADTHSQITAEPLGDDDDVTHLRGSFPGPPGTPYEGGTYKVDI
          	 : . * **: **: **  * ... .     ...:::**:***..**.:** **** :**

MG05478.1 	IIPDTYPFKSPIMKFETKMWHPNVSSQT0GVICLDTLGAGWSPVGTIKMALLSLRMLLES
NCU01225.1	QIPDKYPFKPPSMYLITKIWHPNVSSVT0GAICLDILGTAWSPVGTIKTALLAVRMLLES
FG02029.1 	QIPDQYPFKAPSMRFDTKIWHPNVSSQT0GAICLDTLSSNWSPVQTIKTALLSLRLLLEC
AN2212.1  	KIPTEYPFRPPVMKFITKVWHPNVSSQT0GAICLDTLSSAWSPILTIKAALLSLQSLLST
          	 **  ***:.* * : **:******* * *.**** *.: ***: *** ***::: **. 

MG05478.1 	PNPRDPQDAEVAKMMMEDPDYFKVVAHDWAVRYAGARRTQPPPDQYVRKTP--------V
NCU01225.1	PNPKDPQDAQVAKMLMENPKLFARTAHDWAVKYAGAPRVDKIP---LKYDE--------T
FG02029.1 	PNPKDPQDAEVAKMMMDSPERFAAKAHDWAVQHAGAPRRDVDLS--QYKSE--------G
AN2212.1  	PEPKDPQDAEVATMLLRNPKEFDRVAREWAVQHAGAPRRAAGEGSGGATSESLRELERKE
          	*:*:*****:**.*:: .*. *   *::***::*** *     ..      :  . . . 

MG05478.1 	AAQKAAEDPRR2YCGYNKDLIDRFVSMGFELDKVVEAFLFVGIDRNGGRDYELEEAYMGD
NCU01225.1	AKEGSRNDASR2YAGYNKDLVDRFVNMGFDVEKVVDAFKFVGIDRNGGADYELEEAYMGD
FG02029.1 	APAPPKVDAAR2YMGYNKDLVERFVNMGFPLDNVVEAFIAIGIERNGGRDYVLEEAYMGD
AN2212.1  	KEAREKEDLSK2YDGYNKQLIDRFVHMGFDVDAVVSAFNYYGIDRNGGEDYELEEAYMGD
          	       *  : * ****:*::*** *** :: **.**   **:**** ** ********

MG05478.1 	ITARLLGEQ
NCU01225.1	ITARLLGEQ
FG02029.1 	IVARLLGEQ
AN2212.1  	VTARLLGEP
          	:.******
```
